# A laboratory evaluation of nozzle tip damage in four generations of intraocular lens injector systems using a self-developed damage scale

**DOI:** 10.1038/s41598-022-06696-5

**Published:** 2022-02-17

**Authors:** Hui Fang, Lu Zhang, Sonja Schickhardt, Patrick R. Merz, Weijia Yan, Mélanie Leroux, Gerd U. Auffarth

**Affiliations:** 1 Department of Ophthalmology, David J Apple Center for Vision Research, University Hospital Heidelberg, Im Neuenheimer Feld 400, 69120 Heidelberg, Germany; 2 Université Laval, 2325 Rue de l’Université, Québec, QC G1V 0A6 Canada

**Keywords:** Medical research, Lens diseases

## Abstract

During intraocular lens (IOL) implantation it is not uncommon for the injector’s nozzle-tip to get damaged. However, the damage has not been systematically described or evaluated using an objective scale. In this study we developed our own system—the Heidelberg Score for IOL Injector Damage (“HeiScore”), which was used to grade 60 injectors from four generations of injector models (Monarch III D, AcrySert C, UltraSert, AutonoMe) made by the same manufacturer. (Alcon Laboratories Inc.) HeiScore has six grades of nozzle-tip damage: no damage (which was graded 0); slight scratches (1), deep scratches (2), extensions (3), cracks (4) and bursts (graded number 5). The score for each injector model was the sum of all grades (total number), and we could compare the four injector models. The injectors showed varying damage profiles, from “no damage” to “crack”. A tendency of a lower damage score in the newer generations of IOL injectors was noted. However, a statistically significant difference was observed only between Monarch III D and AutonoMe. The “Heidelberg Score for IOL Injector Damage” could efficiently and effectively evaluate the damage to IOL injector systems, which might help manufacturers optimize the positioning of the IOL in the injector during pre-loading.

## Introduction

A key step in cataract surgery is to deliver the IOL into the capsular bag. Before the 1980s, this step was usually achieved by forceps. In the 1980s, foldable IOLs were developed^1,2^. This innovation allowed lens insertion with subincisions ≤  4 mm^3^, which promoted the use of IOL injectors. In general, IOL injector systems replaced the forceps for IOL implantation.

Complications of IOL delivery associated with IOL injectors include broken haptics, IOL malposition and IOL optic damage^4^. Injectors can produce scratches on the IOL surface^5^. Plastic parts of an injector might be retained in the anterior chamber as a foreign body^6^, which could be a causative agent for toxic anterior segment syndrome (TASS), an acute postoperative sterile inflammatory reaction of the anterior segment tissues to a toxic substance^7,8^. In addition, IOLs might show increased roughness on the optic surface after the IOL delivery and such roughness could be associated with the formation of posterior capsule opacification (PCO)^9^. The IOL experiences friction forces as it passes through the injector nozzle. Examining damage to the injectors after implantation might shed light on how to reduce damage to the IOL, as well as optimize how IOLs should be positioned in the injector during the pre-loading process by the manufacturers.

A few studies^10,11^ have evaluated damage to the IOL injectors after implantation. However, none of these studies measured the damage to injectors in a systematic manner. In this study, we developed a scale—Heidelberg Score for IOL Injector Damage (HeiScore), by which the damage to IOL injectors of four generations from the same manufacturer was examined systematically.

## Results

### Parameters of nozzle tips

**Figure 1:**
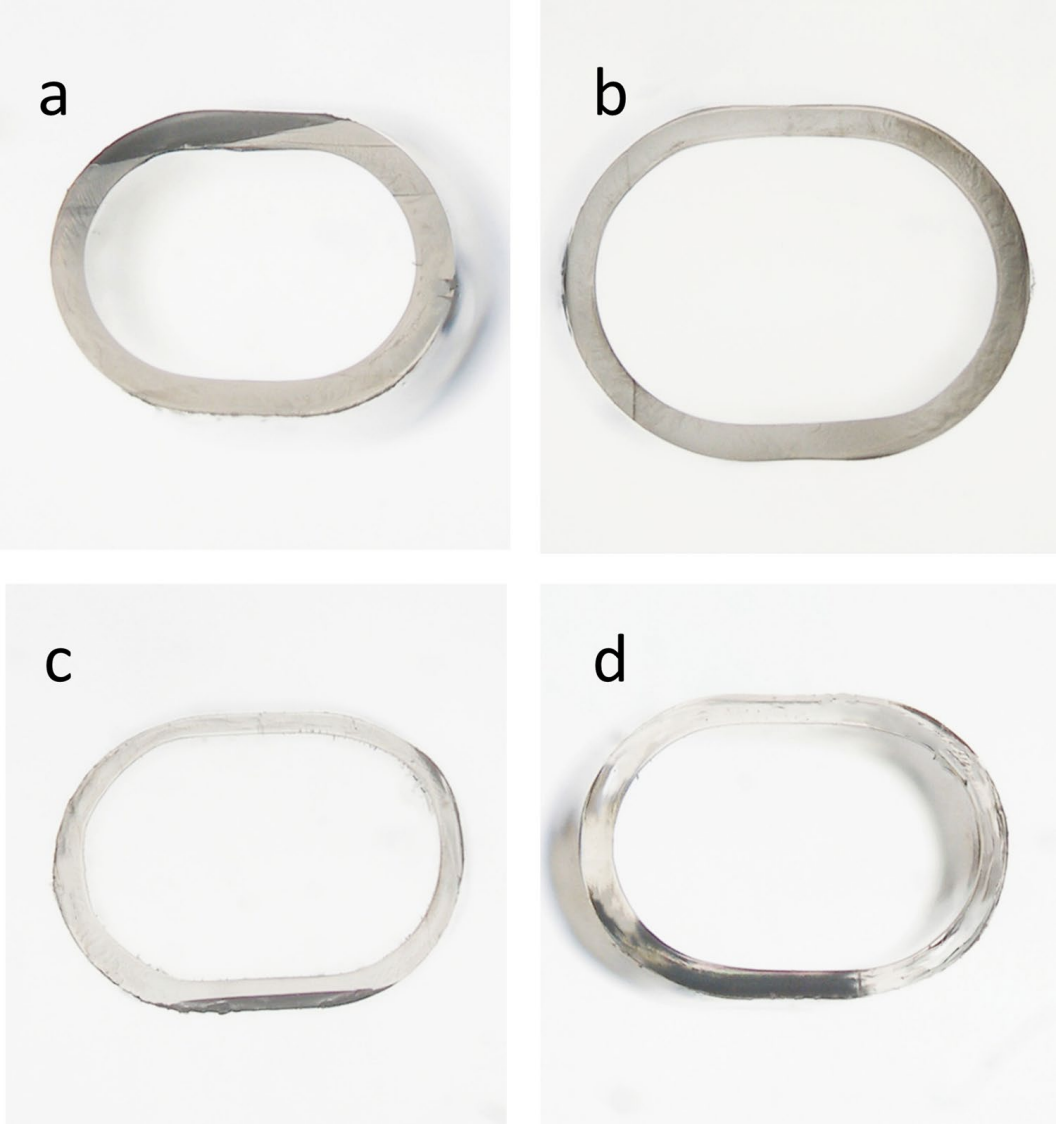
Representative microscopic images of cross-section surfaces for all injector systems (under ×4 magnification). (**a**) Monarch III D Cartridge. (**b**) AcrySert C. (**c**). UltraSert. (**d**). AutonoMe.

The parameters of the nozzle tips for all injector systems are summarized in Table 1. Representative microscopic images of cross-section surfaces for all injector models are shown in Fig. 1.

**Table 1:** Parameters of nozzle tips for all the injector systems.

Injector model	Shape of nozzle tip	Outer cross-section length	Outer cross-section width	Outer cross-section area	Inner cross-section length	Inner cross-section width	Inner cross-section area
		(mm)	(mm)	(mm^2^)	(mm)	(mm)	(mm^2^)
Monarch III D Cartridge	Oval	1.96	1.50	2.44	1.59	1.11	1.54
AcrySert C	Oval	2.23	1.69	3.15	1.91	1.37	2.23
UltraSert	Oval	2.06	1.49	2.59	1.78	1.23	1.87
AutonoMe	Oval	2.05	1.49	2.59	1.75	1.22	1.81

### Distribution of damage profiles

**Figure 2:**
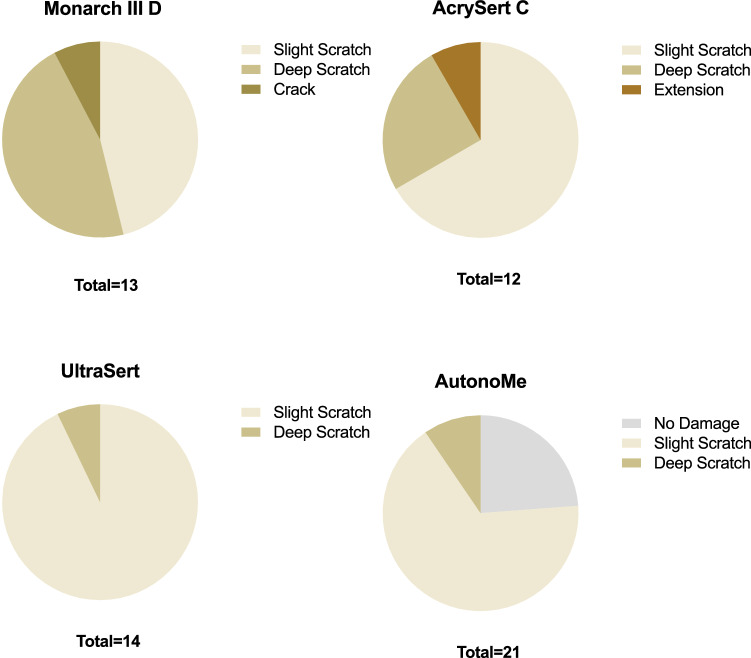
The distribution of damage profile of 4 injector groups.

No gross damage was observed in all the studied IOLs. The distribution of damage profiles of 4 injector groups is shown in Fig. 2. All injector groups in our study presented varying degrees of damage, from “no damage” to “crack”. However, we did not observe grade 5 damage in any of the injector groups.

### Results from statistical analysis

Results of damage score for each IOL injector model are summarized in Table 2. Data were expressed as median (Q1–Q3). Injector Monarch III D Cartridge resulted in the highest value of damage score of 2 (1–2). Statistically significant difference was only observed between injector Monarch III D Cartridge and AutonoMe (*P* < 0.05). No statistically significant difference was observed across 4 groups in terms of the dioptric powers of the IOLs.

**Table 2:** Results of damage score for each injector system.

Injector Model	Damage score		Statistically significant difference (*P* < 0.05, *)			
	Median	Q1–Q3	Monarch III D Cartridge	AcrySert C	UltraSert	AutonoMe
Monarch III D Cartridge	2	1–2				*
AcrySert C	1	1–2				
UltraSert	1	1–1				
AutonoMe	1	0.5–1	*			

## Discussion

In this study, all IOLs were successfully implanted using the injectors without damage to the capsular bag and without any other intraoperative complications. Although the IOL was undamaged, we found varying degrees of damage to the injectors—from no damage to cracked nozzles. To the best of our knowledge, this is the first study presenting a scale system for injector damage after IOL implantation and evaluating injector damage systemically using this scale.

There have been previous studies that evaluated the injector damage after IOL implantation. Wang et al*.*^10^ observed damage such as stress lines and nozzle tip splitting. Stress lines and cracks were also observed by Singh et al.^11^ However, none of these studies graded the damage in a systematic manner.

Damage to the injectors during IOL implantation could be affected by various factors and their interactions with each other, such as the material components of the injectors, the plunger tip geometry, the IOL material and its geometry^9^, as well as the geometry of the nozzle tip including its inner and outer diameters. In our results the Monarch III D had the highest value of damage score. The Monarch plunger is made of metal while the plungers of the other three injectors are made of plastic. The metal plunger might be one factor associated with higher nozzle damage. Metal is harder than plastic and, thus, could cause scratches on the inner walls more easily. In addition, the Monarch has the smallest inner cross-section area, the space within which the IOL passes through during implantation. If the space is too small, greater friction and more damage to the injector nozzle could be anticipated.

Although there was statistically significant difference only between the Monarch III D and the AutonoMe, we did observe a tendency of smaller damage score in the newer generations of IOL injector models. As one study^10^ has pointed out, the special “depth guard” design of injector UltraSert increases the mechanical strength of the nozzle, making such injectors more resistant to splitting. Compared to the earlier generations, the latest injector, the AutonoMe is characterized by not only the “depth guard” nozzle tip design, but also a unique automated delivery design. Compared to screw-type or syringe-type design, the automated delivery manner provides more consistent and predicable implantation, decreasing the probability of damage to the nozzle tip due to a sudden release of the IOL.

The dioptric power of all IOLs included in our study ranged from + 15D to + 25D, which is the most commonly used diopter range in clinical practice. No statistically significant difference was observed across four groups in terms of the dioptric power of IOLs. Besides, all injectors inspected in this study were adopted based on the recommended range of IOL diopters (Supplemental Table 1). Thus, the effect of different diopters of the IOLs is negligible when the injectors are maneuvered according to manufacturer’s instructions. The incision sizes in our study were either 2.4 mm or 2.5 mm clear corneal incisions, which guaranteed that no extra friction to nozzle tips was generated due to too tight or too small incision sizes (Supplemental Table 1).

This study is not without limitations. First, although our scale system was based on massive data of injector damage, the possibility of not covering all types of damage could not be ruled out. Second, this study was a retrospective study and the main purpose of the study was to introduce our scale system and raise awareness of different extent of damage to the injectors. More studies are still warranted to explore the correlation between the extent of injector damage and its clinical impact.

In conclusion, our “Heidelberg Score for IOL Injector Damage” could make it possible to evaluate systematically and categorize the injector damage, as well as to optimize the IOLs positioning within the injectors. A tendency of less nozzle damage in the newer generations of IOL injector systems was observed in our study. However, additional improvements in the designs and materials are still required to further alleviate the damage and provide smoother and more predictable IOL implantation.

## Materials and methods

### IOL injector models (Supplemental Table 1)

- Monarch III D. The oldest injector model in our study that was introduced in 2007. It is a system of a metal injector with a plastic cartridge for holding the IOL which is manually loaded in the operating room prior to surgery.
- AcrySert C. Introduced in 2010. Unlike Monarch, the IOL was loaded by the manufacturer before packaging, known as pre-loaded injectors.
- UltraSert. Introduced in 2015. “Depth guard” design was first adopted in this model.
- AutonoMe. Introduced in 2017. It is the first automated pre-loaded IOL delivery model with a CO_2_-powered delivery mechanism.

**Figure 3:**
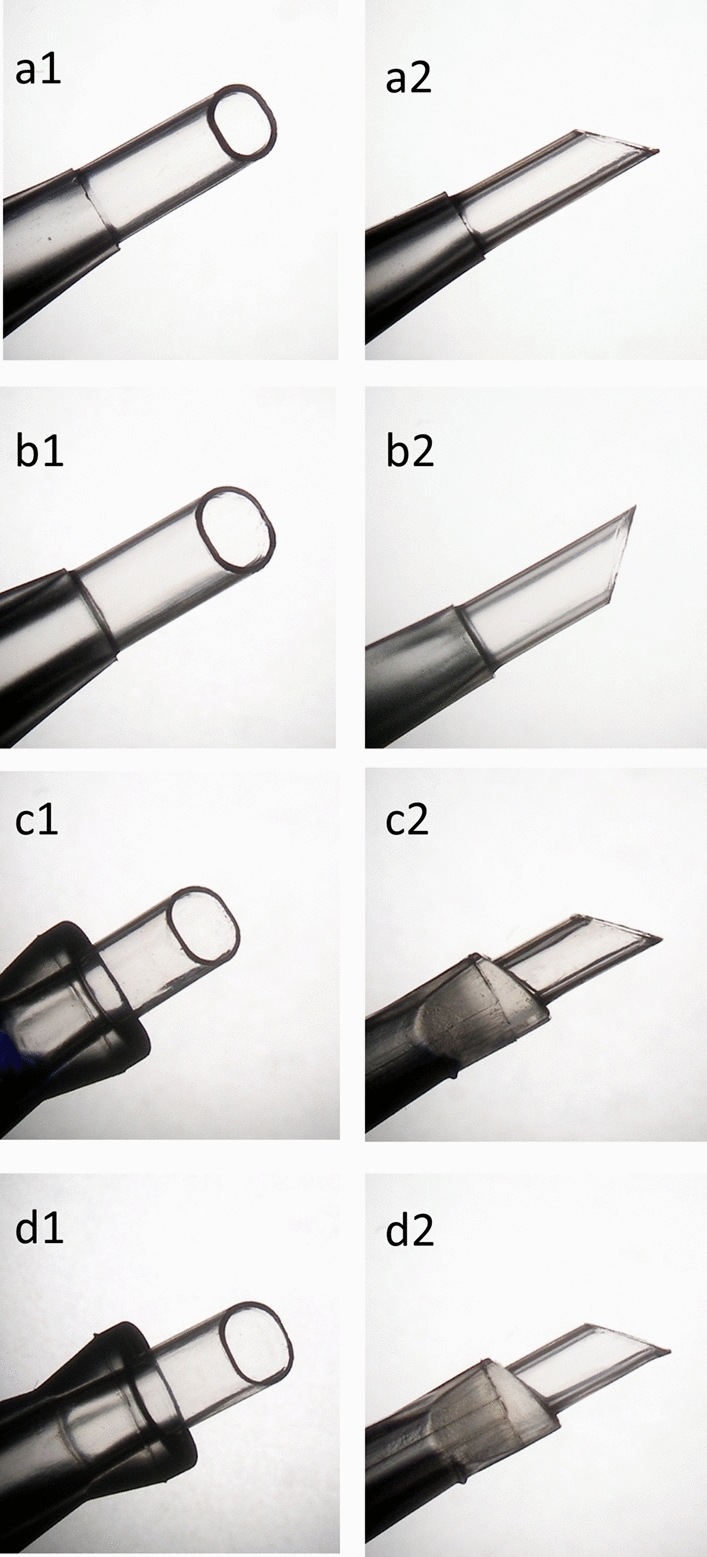
Representative microscopic images of 4 unused IOL injector systems. (**a1**,**a2**) Axial view and profile view of Monarch III D Cartridge. (**b1,b2)** Axial view and profile view of AcrySert C. (**c1,c2**) Axial view and profile view of UltraSert. (**d1,d2**) Axial view and profile view of AutonoMe.

Sixty IOL injectors of four models from the same manufacturer (Alcon Laboratories Inc., Fort Worth, Texas, USA) (Supplemental Table 1) were used in a series of routine, uncomplicated cataract operations for IOL delivery at the Heidelberg University Eye Hospital. All surgical operations were performed by the same experienced surgeon (GUA). Representative microscopic images of four unused IOL injectors are shown in Fig. 3. The IOL powers ranged from + 15D to + 25D. The incisions were either 2.4 mm or 2.5 mm clear corneal incisions. In all cases, the injector was primed with ophthalmic viscosurgical device (OVD) of 1% sodium hyaluronate (ProVisc, Alcon Laboratories Inc., Fort Worth, Texas, USA). The lens was injected into the eyes following the manufacturer’s instructions. The injector force used for injection was appropriate and necessary. In order to obtain a cross section surface, we cut all four nozzle tips, a the position where the bevel angle commences. Photographs of the cross-section surfaces were taken under the microscope (Olympus BX50, Olympus K. K.). Parameters (*i.e*., length and width, etc.) of the cross-section surface were measured using a ruler under the microscope as a standard to calibrate the measurements. Image J software (version 1.52a, NIH, Bethesda, Maryland, USA) was used to measure the parameters of the cross-section surface of the nozzle tips. Inner cross-section areas were also calculated using Image J software.

### Heidelberg score for IOL injector damage (HeiScore)

According to our system of scoring, the damage observed on the injectors could be classified into the following six grades.

*Grade 0* There is no damage observed on the nozzle tips.

*Grade 1 *There is slight scratch—fine stress lines on the inner tube or/and slight discontinuity at the nozzle tips.

*Grade 2* There is deep scratch—deep stress lines on the inner tube or/and obvious discontinuity at the nozzle tips.

*Grade 3* There is extension of “deep stress line”, but the deep stress line does not reach the level of full thickness tube crack.

*Grade 4* There is crack - full thickness crack of the injector tubes.

**Figure 4:**
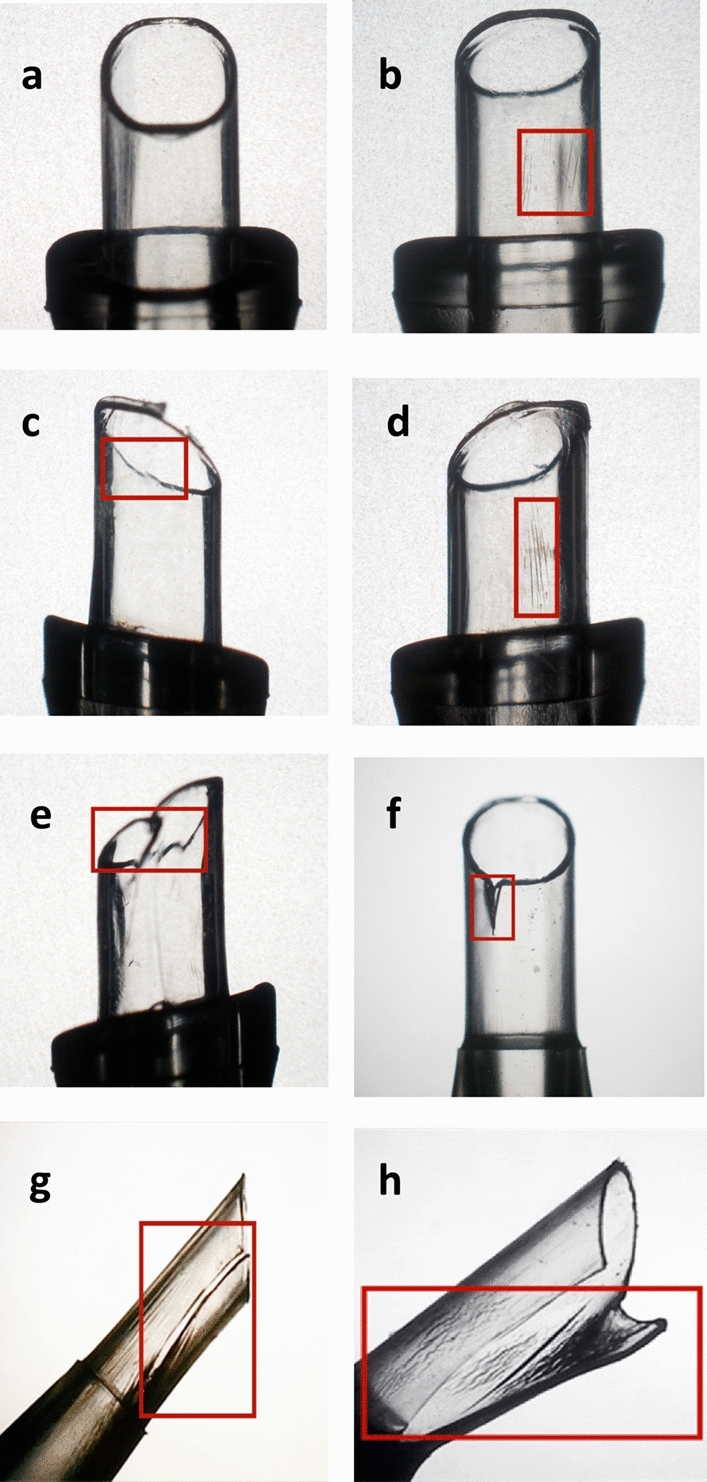
Representative microscopic images of each damage scale (under ×4 magnification). (**a**) No damage. (**b**) Red square indicates fine stress lines on the inner tube, graded as “slight scratches”. (**c**) Red square indicates slight discontinuity at the nozzle tip, graded as “slight scratches”. (**d**) Red square indicates deep stress lines on the inner tube, graded as “deep scratches”. (**e**) Red square indicates obvious discontinuity at the nozzle tip, graded as “deep scratches”. (**f**) Red square indicates partial crack of inner tube, graded as “extension”. (**g**) Red square indicates full thickness of inner tube crack, graded as “crack”. (**h**) Red square indicates burst of the nozzle tube, graded as “burst”.

*Grade 5* There is burst of the injector tubes. Representative microscopic images of each damage grade are shown in Fig. 4. Each damage grade was assigned a score from 0 to 5 (Table 3).

**Table 3:** Corresponding score to each damage scale.

Damage scale	Score
No damage	0
Slight scratch	1
Deep scratch	2
Extension	3
Crack	4
Burst	5

### Damage evaluation of the nozzle tips

At the end of each implantation, a gross examination was done under the microscope to detect if there was any damage to the IOL. After each surgery session, the used injectors were collected from the operation room and sent to our laboratory. The nozzles were immersed in the distilled water for ten minutes to remove any residual OVD, and subsequently left to air-dry. Care was taken to avoid damaging the injector nozzle tips while handling. After being air-dried, the nozzles were inspected using an optical microscope (Olympus BX50, Olympus K.K.). Each nozzle tip was first inspected in the “bevel-down” and “bevel-up” orientation, followed by both of the lateral orientations. Photographs were taken at each orientation under ×1.25 and ×4 magnifications, respectively.

### Statistical analysis

The Saphiro-Wilks test was performed to detect nonparametric distributions in damage scores and dioptric powers of IOLs. The Kruskal–Wallis *H* test with Bonferroni adjustment for post-hoc comparison was used to examine the significant differences between groups. SPSS (Version 26.0, IBM, NY, USA) was used for all statistical analyses, and a resultant *P* value less than 0.05 was chosen for statistical significance.

## Supplementary Information

Supplementary Information.

## Data Availability

The data used to support the findings in this study are available from the corresponding author upon reasonable request.
