# Supplementary material for: A laboratory evaluation of nozzle tip damage in four generations of intraocular lens injector systems using a self-developed damage scale
Source: Sci Rep. 2022 Feb 17;12:2723. doi: 10.1038/s41598-022-06696-5 (PMC8854687; doi:10.1038/s41598-022-06696-5)
Supplement: Supplementary file 1 — Supplementary Information. [file 41598_2022_6696_MOESM1_ESM.pdf]

A laboratory evaluation of nozzle tip damage in four generations of intraocular lens injector systems using a self-developed damage scale

Hui Fang, MS[1,†], Lu Zhang, MS[1,†], Sonja Schickhardt, PhD[1], Patrick R. Merz, PhD[1], Weijia Yan, MS[1], Mélanie Leroux[2], Gerd U. Auffarth, MD, FEBO[1,*]

Supplemental Table 1. Test injectors from the same manufacturer

Injector Model	IOL Model	Total Number(n)	Loading Method	Recommended Dioptric Range (diopter)	Smallest Incision Size
Monarch Ⅲ D Cartridge	SN60WF	13	Manually loaded	+6D - +27D	2.4
AcrySert	SN6CWS	12	Pre-loaded	+6D - +30D	2.2
UltraSert	AU00T0	14	Pre-loaded	+6D - +30D	2.2
AutonoMe	Clareon	21	Pre-loaded	+6D - +30D	2.2
